# Identification of tumor microenvironment-related prognostic genes in colorectal cancer based on bioinformatic methods

**DOI:** 10.1038/s41598-021-94541-6

**Published:** 2021-07-22

**Authors:** Yi Liu, Long Cheng, Chao Li, Chen Zhang, Lei Wang, Jiantao Zhang

**Affiliations:** 1 Department of Colorectal and Anal Surgery, The First Hospital of Jilin University, Changchun, Jilin People’s Republic of China; 2 Department of Orthopedics, The First Hospital of Jilin University, Changchun, Jilin People’s Republic of China

**Keywords:** Cancer microenvironment, Computational biology and bioinformatics

## Abstract

Colorectal cancer (CRC) ranks fourth among the deadliest cancers globally, and the progression is highly affected by the tumor microenvironment (TME). This study explores the relationship between TME and colorectal cancer prognosis and identifies prognostic genes related to the CRC microenvironment. We collected the gene expression data from The Cancer Genome Atlas (TCGA) and calculated the scores of stromal/immune cells and their relations to clinical outcomes in colorectal cancer by the ESTIMATE algorithm. Lower immune scores were significantly related to the malignant progression of CRC (metastasis, p = 0.001). We screened 292 differentially expressed genes (DEGs) by dividing CRC cases into high and low stromal/immune score groups. Functional enrichment analyses and protein–protein interaction (PPI) networks illustrated that these DEGs were closely involved in immune response, cytokine–cytokine receptor interaction, and chemokine signaling pathway. Six DEGs (FABP4, MEOX2, MMP12, ERMN, TNFAIP6, and CHST11) with prognostic value were identified by survival analysis and validated in two independent cohorts (GSE17538 and GSE161158). The six DEGs were significantly related to immune cell infiltration levels based on the Tumor Immune Estimation Resource (TIMER). The results might contribute to discovering new diagnostic and prognostic biomarkers and new treatment targets for colorectal cancer.

## Introduction

Colorectal cancer (CRC) accounts for about 10 percent of all cancers diagnosed and nearly 900,000 deaths a year^1^. As developing countries continue to improve, there will be 2.5 million new colorectal cancer cases in 2035^2^. Treatment strategies for CRC include endoscopic therapy, surgical treatment, metastatic therapy, radiotherapy, and chemotherapy. Since tumors only show symptoms at a late stage, it is essential to identify reliable prognostic biomarkers to provide new ideas for exploring immunotherapeutic targets to improve survival in CRC patients.

The tumor microenvironment (TME) is a complex system that affects tumor growth and development^3^. It is composed of tumor cells, stromal cells, immune cells, and extracellular matrix. Immune cells and stromal cells are the two main non-tumor components besides tumor cells^4^. In recent years, many studies have shown that the tumor microenvironment significantly affects tumor growth and progression and offers potential value in the diagnosis and prognosis prediction of tumors^5–10^. Moreover, it has been reported that the tumor microenvironment highly influences the progression of colorectal cancer^5,11^. Recently, with the rapid development of precision medicine, more and more researchers are using statistical algorithms to explore new diagnostic and therapeutic targets of cancer. The Cancer Genome Atlas (TCGA) provides a comprehensive genome map and detailed clinical and follow-up information, making it suitable for studying the association between tumor immunity and genomic characteristics^12^. ESTIMATE (estimation of stromal and Immune cells in malignant tumor tissues using expression data) algorithm is a tool for calculating the scores of stromal and immune cells in malignant tumors, which is developed by Yoshihara et al.^13^. It has been proved that the prediction method has good accuracy and practicability on independent datasets. Therefore, this study aimed to identify the TME related genes affecting the prognosis of colorectal cancer through the TCGA database. We also assessed the association of these genes with infiltrating immune cells in the colorectal cancer microenvironment.

## Materials and methods

### Data collection

We gathered gene expression data and clinical information of colorectal cancer (CRC) patients from the TCGA database (https://portal.gdc.cancer.gov/). There are RNA sequencing datasets of 568 colorectal cancer tissues. The clinical information included age, gender, futime (overall survival time), fustate (survival status), stage, and TNM status. After the CRC patients with a survival time of fewer than 30 days were excluded, only 512 patients remained and were chosen for the analysis (S1). We applied the ESTIMATE algorithm to calculate the immune score and stromal score of CRC patients. The GSE17538 data set (n = 229, S2) and GSE161158 data set (n = 188) were downloaded from the GEO database (https://www.ncbi.nlm.nih.gov/geo/) as external validation series. Based on identical futime, fustat, age and stage, there are sixty-two overlapping patients between the two external validation cohorts. We excluded the sixty-two overlapping patients in the GSE161158 cohort. Therefore in the GSE161158 cohort, only 126 patients were included in the study (S3). The microarray data of GSE17538 and GSE161158 were based on the Affymetrix Human Genome U133 Plus 2.0 Array platform. We applied the Tumor Immune Estimation Resource (TIMER)^14^ (https://cistrome.shinyapps.io/timer/) to explore the relationship between DEGs expression and immune cell infiltration level. We used the human protein atlas (HPA) database (http://www.proteinatlas.org/) to verify the translation level of prognostic genes related to the tumor microenvironment.

### Expression analysis of differentially expressed genes (DEGs)

Based on the median stromal/immune score, we categorized CRC samples in the TCGA database into high and low stromal/immune score groups for further research. Using the limma package^15^ and Wilcox or Kruskal.test. Test in R software (version 4.0.3, https://www.r-project.org/), we selected the differentially expressed genes between the high/low immune score group. We similarly analyzed the stromal group. The cut-off value is log fold change (FC) > 1.5 and false discovery rate (FDR) < 0.05. The R packages ggplot2 and pheatmap were used for the generation of heatmaps. The DEGs were displayed by the R package VennDiagram.

### GO analysis, KEGG analysis, and PPI

The GO function analyses consisted of biological processes (BP), cellular components (CC), and molecular function (MF). Kyoto Encyclopedia of Genes and Genomes (KEGG) pathway analysis explores significant pathways related to DEGs. The R packages “cluster profile” (version 3.17.0), “org.Hs.eg.db” (version 3.11.1), “enrichplot” (version 1.8.1), and “ggplot2” (version 3.3.0) were used to conduct function enrichment analyses with a cut-off criterion of p < 0.05. We used an open-source software platform, Search Tool for the Retrieval of Interacting Genes (STRING, https://string-db.org/)^16^, to build the PPI network of DEGs. We selected the degree of interactions between proteins with a combined score > 0.9 and performed analysis using Cytoscape (https://cytoscape.org/; version 3.7.1)^17^. We then used the MCODE^18^ plug-in in the Cytoscape software to conduct modular analysis and selected the top two significant modules with more than five nodes for further analysis. We used Metascape (http://metascape.org/gp/index) to conduct enrichment analysis of module genes, which is an effective and efficient tool to provide a comprehensive gene list annotation in the big data era^19^.

### Survival analysis

To identify whether the TME-related DEGs relate to the prognosis of CRC patients, we performed Kaplan–Meier survival analysis using the R package "survival". We divided the patients into two groups for survival analysis according to the median value of the expression of each gene. And we verified the prognostic DEGs in the GSE17538 and GSE161158 data sets. For all results, p < 0.05 was considered statistically significant.

## Results

### Lower immune scores significantly related to malignant progression of CRC

**Figure 1:**
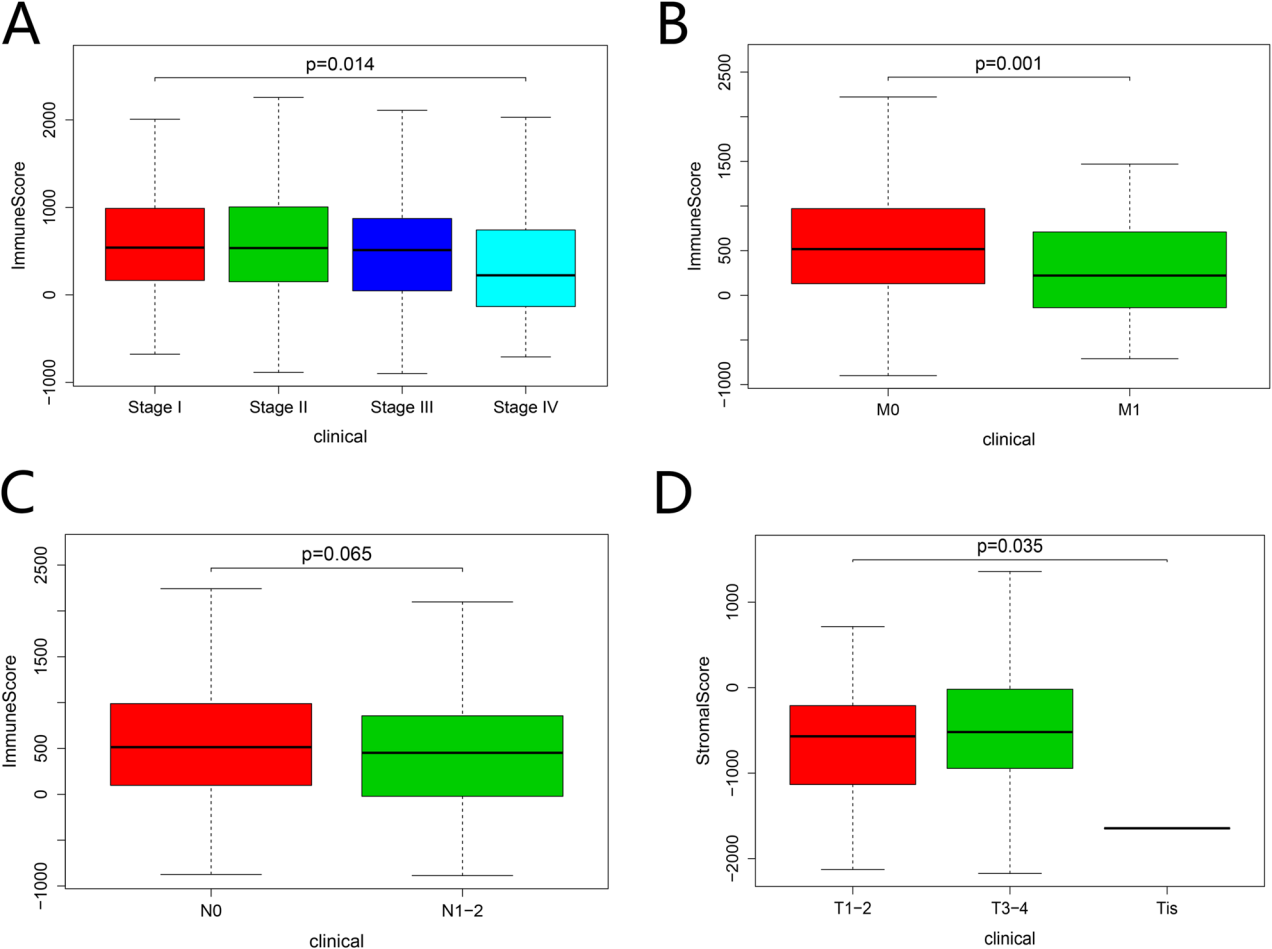
Lower immune scores are significantly related to malignant progression of CRC. The histogram revealed the distribution of immune scores in CRC-stage (p = 0.014) (**A**), CRC-M (p = 0.001) (**B**), CRC-N (p = 0.065) (**C**). Lower immune scores were associated with stage and tumor metastasis cases. The distribution of stromal scores in CRC-T (p = 0.035) (**D**). High-stromal score was associated with tumor size.

We collected gene expression profile data and clinical information of 568 CRC patients from the TCGA data portal. We calculated that stromal scores ranged from -2173.94 to 1941.04 and immune scores from -952.87 to 2979.35. We conducted the difference analysis of immune/stromal scores according to different clinical characteristics. As shown in Fig. 1A–C, American CRC patients' immune scores decreased with increasing tumor metastasis, but it has no significant relationship with the size of the primary tumor. Besides, we found that stromal scores increased gradually as the T stage increased Fig. 1D. The correlation between immune/stromal scores and other clinical traits is in Fig. S1. The results indicated that stromal/immune scores might be helpful indicators to reflect the malignant degree of CRC.

### Expression analysis of differentially expressed genes (DEGs)

**Figure 2:**
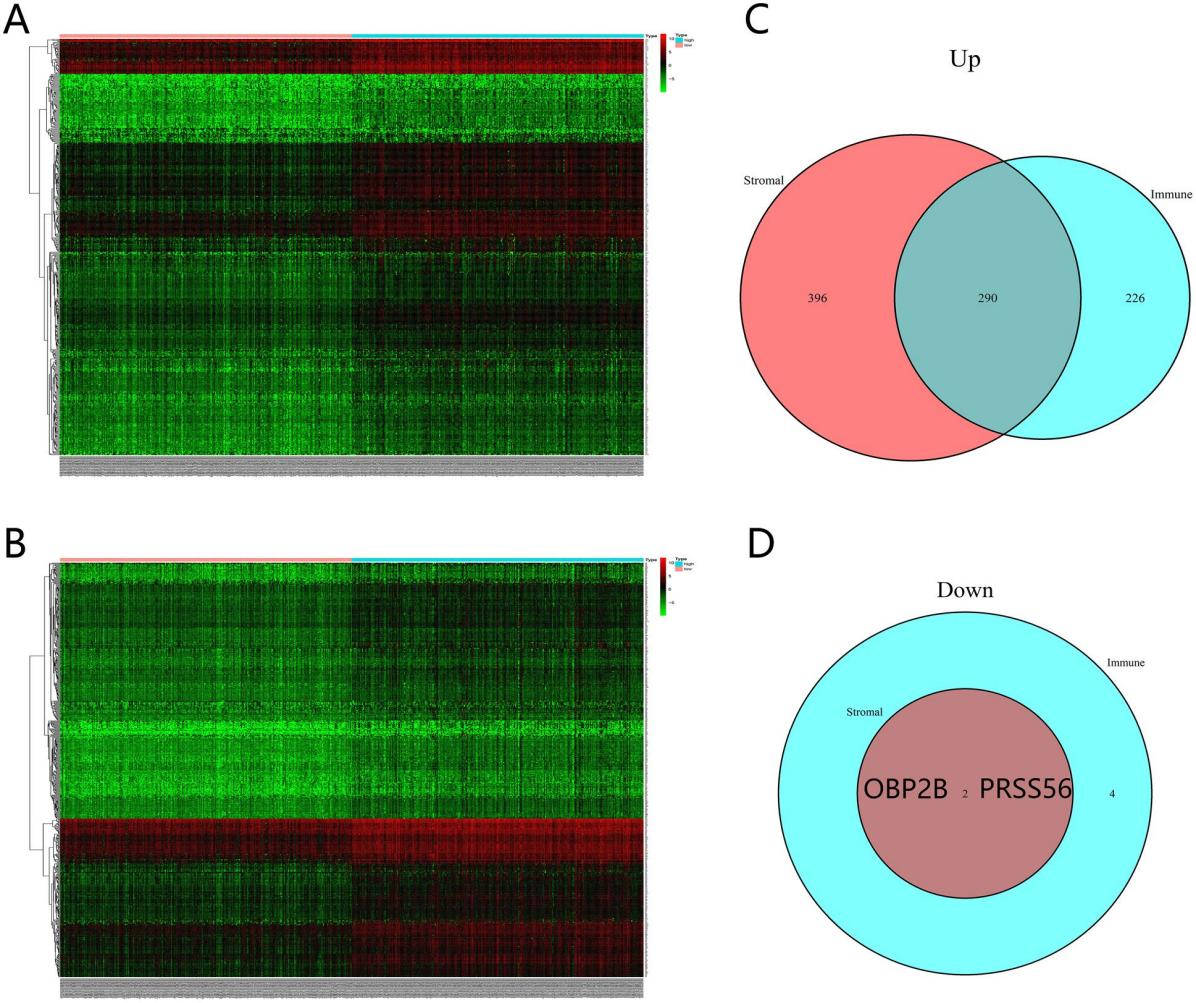
Analysis of gene expression profiles with immune scores and stromal scores. Heatmaps show differentially expressed gene profiles between high and low immune scores/stromal scores groups. (**A** Immune scores, (**B**) Stromal scores, low score in left and high score in the right; Red represents higher expression genes, and green represents lower expression genes, black represents same expression genes; |log2fold-change| > 1.5, FDR < 0.05). Venn plots revealed 292 intersect genes, including 290 upregulated genes in higher immune/stromal scores (**C**) and 2 down-regulated genes in lower immune/stromal scores (**D**).

We divided the CRC patients into high- and low-stromal/immune scores groups following median stromal (− 527.66) scores and immune (501.24) scores. We then ascertained the differentially expressed genes (DEGs) by calculating gene expression data between high and low immune/stromal scores groups using the limma package. The heatmaps of immune scores (Fig. 2A) and stromal scores (Fig. 2B) displayed the gene expression profiles of CRC samples distinctly. We demonstrated 516 upregulated DEGs, and 6 downregulated DEGs based on the immune scores (|log2fold-change| > 1.5, FDR < 0.05, S4). And 686 upregulated DEGs, and 2 downregulated DEGs were screened based on stromal scores (|log2fold-change| > 1.5, FDR < 0.05, S5). Finally, we ascertained 292 intersect genes for further analysis, with 290 upregulated and 2 downregulated genes (Figs. 2C,D).

### GO analysis, KEGG analysis, and PPI

**Figure 3:**
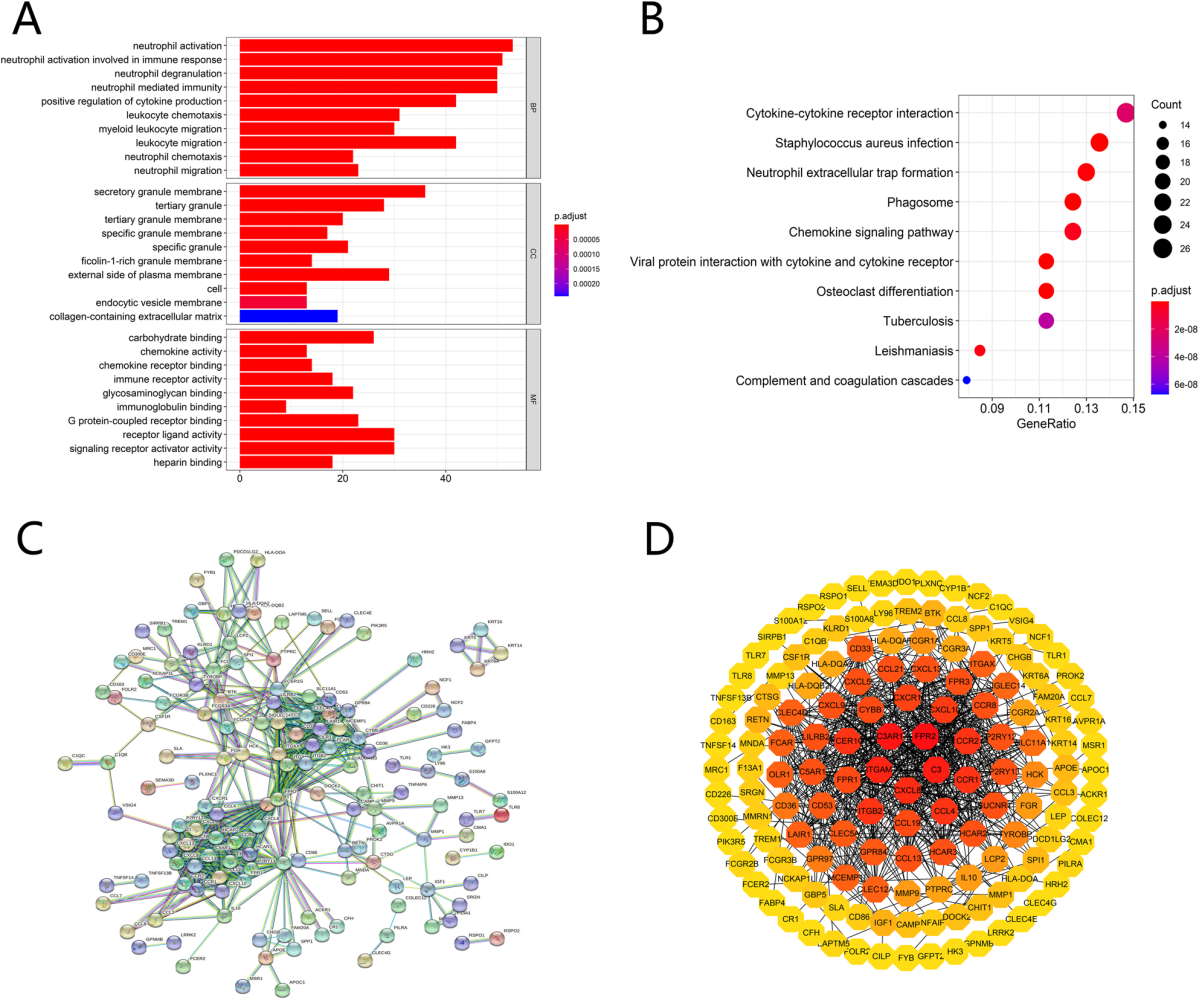
GO functional enrichment analyses, KEGG pathway analyses, and PPI network of DEGs. (**A**). The top 30 significantly enriched GO terms, including biological process (BP), molecular function (MF), and cellular component (CC). (**B**). KEGG pathway analyses of DEGs, The top 10 pathway enrichment was shown. The PPI network of the TME-related DEGs (**C**, **D**). [STRING, https://string-db.org/; Cytoscape (https://cytoscape.org/; version 3.7.1)].

**Figure 4:**
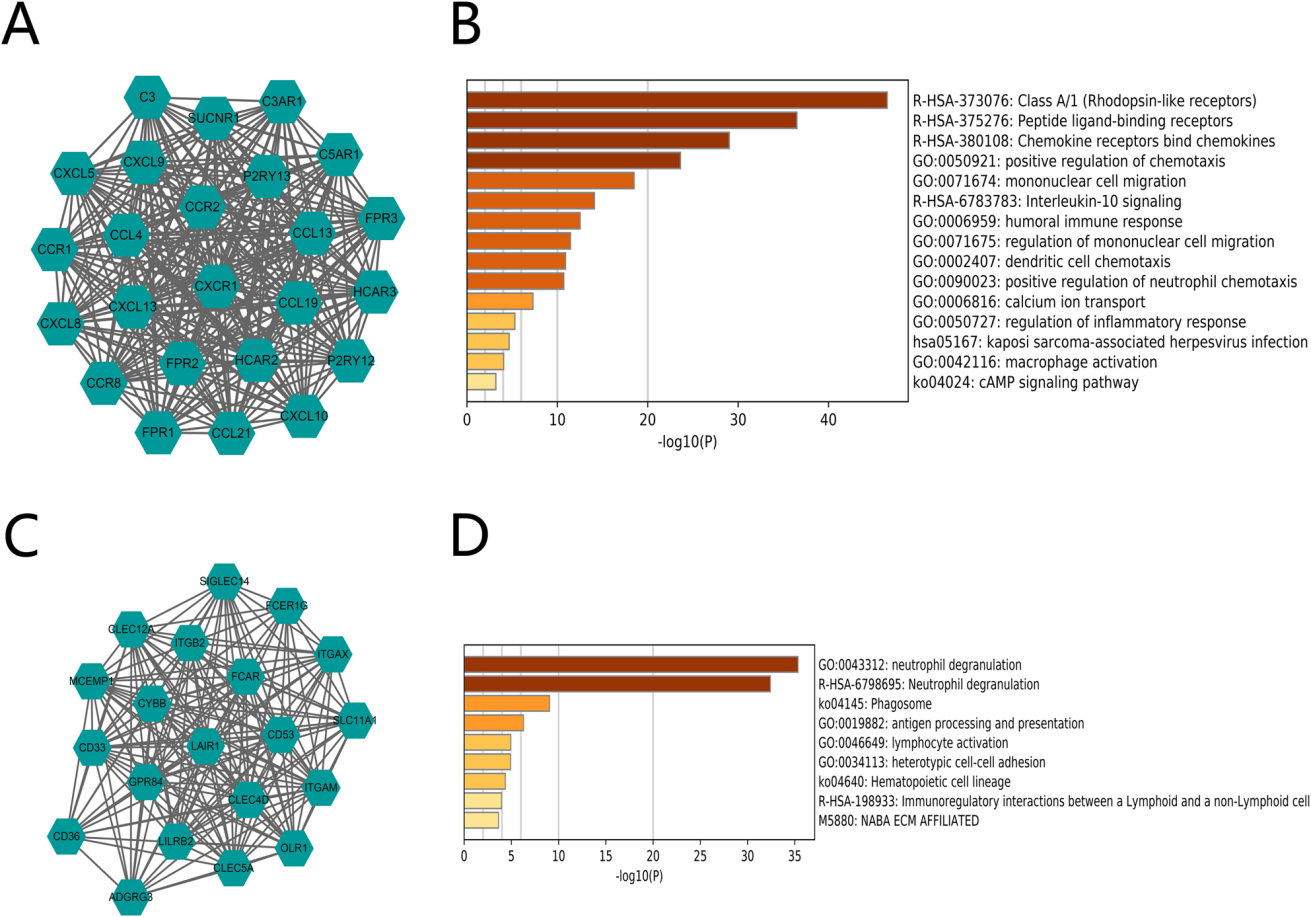
The top two significant modules with more than 5 nodes. (**A**) PPI network of the 24 TME-related DEGs in Module 1. (**B**) Functional enrichment analysis of module 1. (**C**) PPI network of the 20 TME-related DEGs in Module 2. (**D**) Functional enrichment analysis of module 2. (Cytoscape (https://cytoscape.org/; version 3.7.1); Metascape (http://metascape.org/gp/index)).

To investigate the biological functions of these DEGs, we implemented functional enrichment analysis of the 292 intersect genes. The GO analysis indicated that DEGs were mainly concentrated in neutrophil activation involved in immune response, leukocyte migration, secretory granule membrane, receptor-ligand activity, immunoglobulin binding, signaling receptor activator activity, and immune receptor activity (Fig. 3A). The KEGG pathways were displayed in Fig. 3B, mainly enriched in cytokine–cytokine receptor interaction, phagosome, and chemokine signaling pathway. To further explored the potential relationship between the DEGs, we conducted a protein–protein interaction (PPI) network using the STRING tool with the minimum required interaction score > 0.900 (Fig. 3C). The Cytoscape software was used to reconstruct the PPI network. The network is formed from eight modules, including 145 nodes and 701 edges (Fig. 3D). We selected the top two significant modules with more than 5 nodes for further analysis. Module1, contains 24 nodes and 276 edges (Fig. 4A). The Metascape gene list analysis indicated that Module 1 was mainly involved in humoral immune response, dendritic cell chemotaxis, neutrophil chemotaxis, peptide ligand-binding receptors, and cAMP signaling pathway (Fig. 4B). Module 2 contains 20 nodes and 180 edges (Fig. 4C). The enrichment analysis revealed the 20 genes were mainly associated with neutrophil degranulation, antigen processing and presentation, hematopoietic cell lineage, lymphocyte activation, and immunoregulatory interactions between a Lymphoid and a non-Lymphoid cell (Fig. 4D). The results further illustrated that these DEGs were closely associated with TME and immune response.

### Mining and verification of DEGs with predictive value

**Figure 5:**
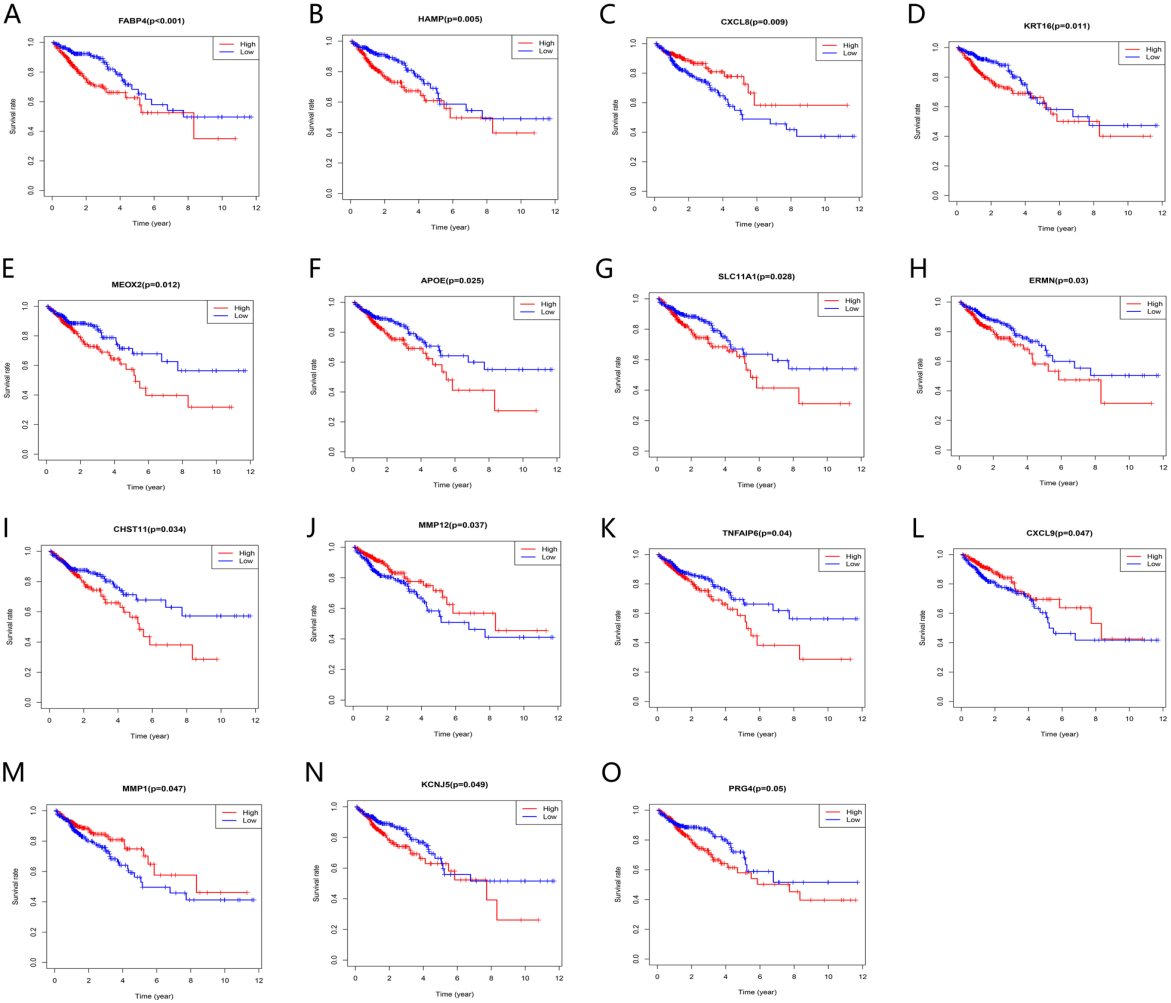
Association between the TME-related DEGs expressions and overall survival in TCGA. Fifteen genes were found to be related to CRC patients’ survival. p < 0.05.

**Figure 6:**
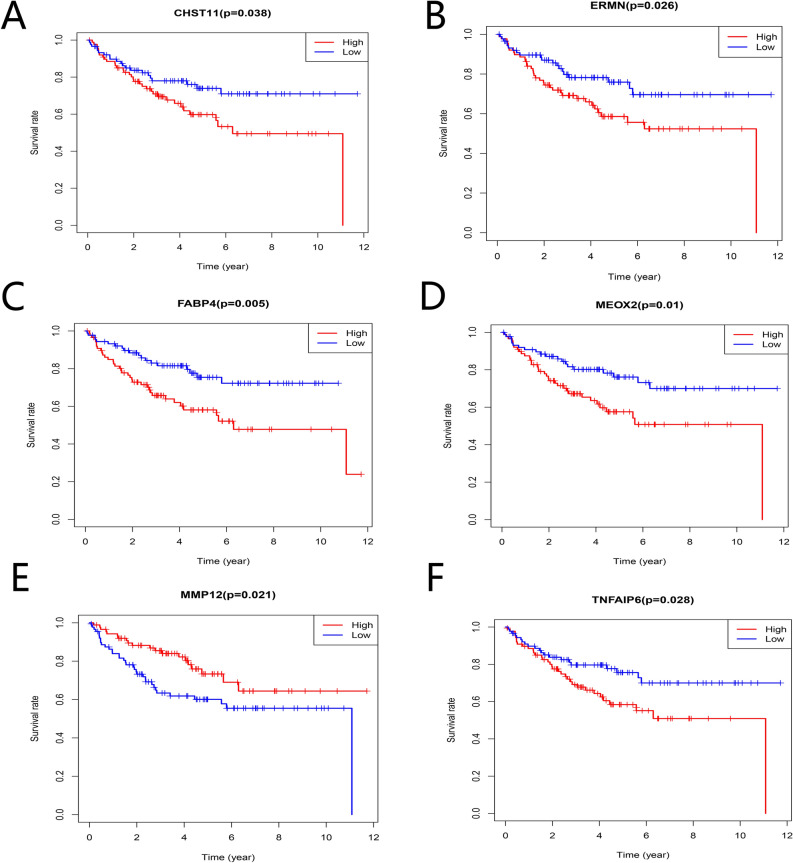
Validation of correlation of DEGs associated with prognosis extracted from TCGA database with overall survival in GSE17538 of GEO database. Six genes (MMP12, CHST11, ERMN, FABP4, MEOX2, and TNFAIP6) were verified to be related to prognosis. p < 0.05.

**Figure 7:**
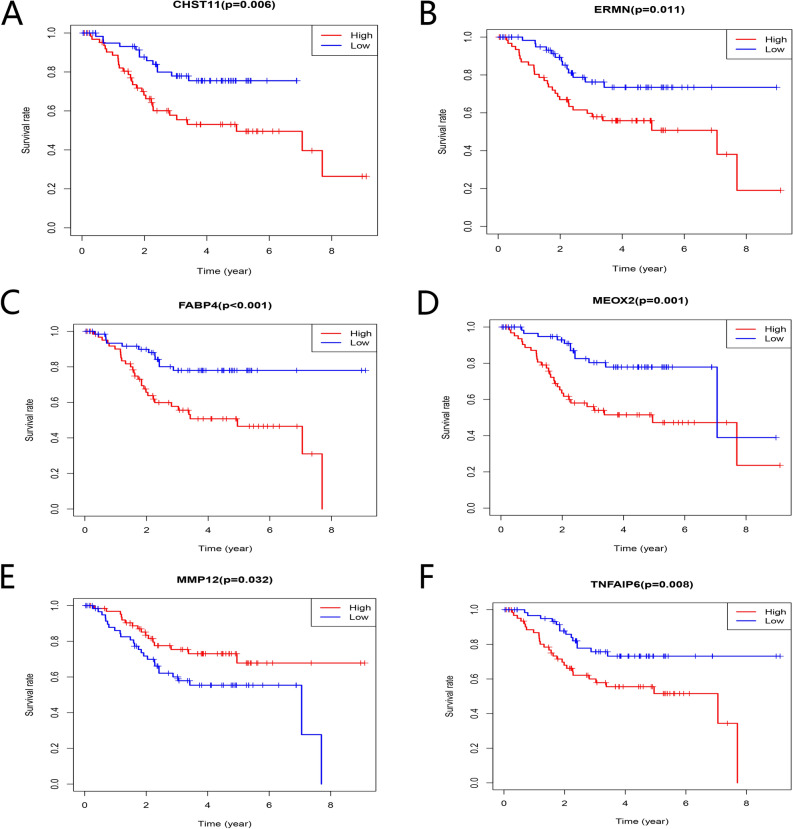
Validation of correlation of DEGs associated with prognosis extracted from TCGA database with overall survival in GSE161158 of GEO database. Six genes (MMP12, CHST11, ERMN, FABP4, MEOX2, and TNFAIP6) were verified to be related to prognosis. p < 0.05.

**Figure 8:**
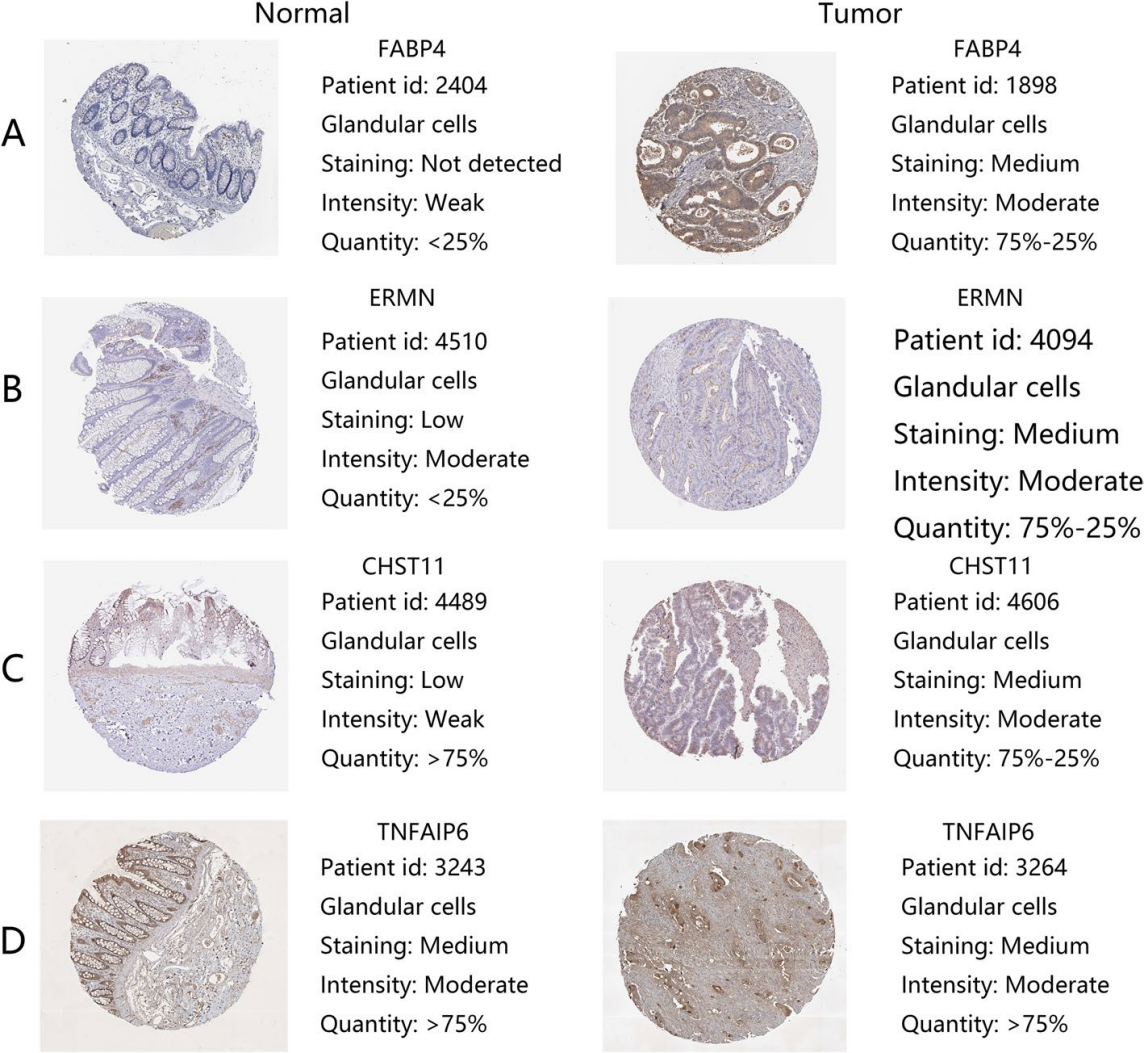
Immunohistochemistry analysis for FABP4 (**A**), ERMN (**B**), CHST11 (**C**), and TNFAIP6 (**D**) in colorectal cancer (HPA database, http://www.proteinatlas.org/).

The Kaplan–Meier survival analysis showed that 15 TME-related DEGs were significantly related to the prognosis of colorectal cancer (Fig. 5, p < 0.05). As shown in the figure, the expression levels of some genes were positively correlated with prognosis, while some genes were negative. Finally, to verify whether the 15 prognostic-related genes have prognostic significance in other independent databases, we downloaded and analyzed the GSE17538 and GSE161158 data sets from the GEO database. The results showed that six TME-related DEGs were associated with the overall survival rates of colorectal cancer patients **(**Figs. 6, 7, p < 0.05). And the higher expression of MMP12 (Fig. 6E, p = 0.021; Fig. 7E, p = 0.045) predicts better prognosis, while CHST11 (Fig. 6A, p = 0.038; Fig. 7A, p < 0.001), ERMN (Fig. 6B, p = 0.026; Fig. 7B, p = 0.006), FABP4 (Fig. 6C, p = 0.005; Fig. 7C, p = 0.003), MEOX2 (Fig. 6D, p = 0.01; Fig. 7D, p = 0.002) and TNFAIP6 (Fig. 6F, p = 0.028; Fig. 7F, p = 0.002) predicted worse prognosis. In addition to studying the differences in the mRNA expression levels of the six DEGs in colorectal cancer patients, we also used the HPA database to explore the expression levels of the proteins encoding by the genes (Fig. 8). The results indicated that FABP4, ERMN, and CHST11 were overexpressed in CRC tumor tissues compared with normal tissues. However, there was no significant difference in the expression of TNFAIP6 between CRC tumor tissues and normal tissues. The protein level of MEOX2 was not detected in normal tissues and cancer tissues of the intestine, and the HPA database did not provide immunohistochemical information of MMP12.

### Association of tumor-infiltrating immunocyte fraction with the 6 DEGs

**Figure 9:**
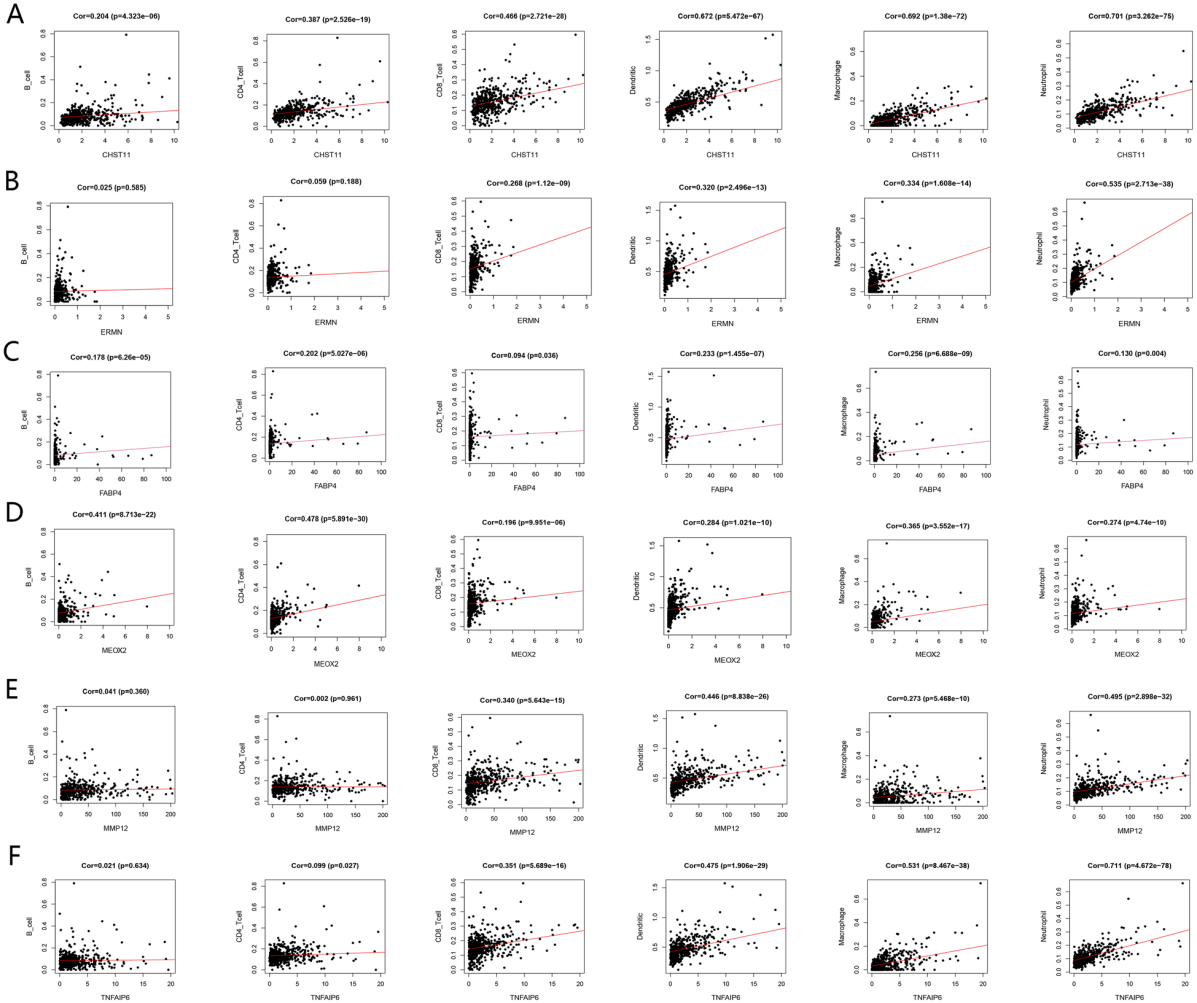
Correlation analysis between the TME-related prognostic DEGs (MMP12, CHST11, ERMN, FABP4, MEOX2 and TNFAIP6) and infiltration levels of B cell, CD8 + T cell, CD4 + T cell, macrophage, neutrophil, and dendritic cell.

To further clarify the relationship between the expression of the identified DEGs and the immune infiltration of the CRC tumor microenvironment, we used the TIMER for exploratory analysis. The immune cells include B cell, CD4 + T cell, CD8 + T cell, macrophage, neutrophil, and dendritic cell in our research. As shown in Fig. 9, the expression of the six identified DEGs were positively correlated with the level of infiltration of different immune cells. These results indicated that the six prognostic-related DEGs might regulate the CRC immune microenvironment by affecting the infiltration of immune cells.

## Discussion

This study calculated the scores of stromal/immune cells and their relations to prognosis and clinical outcomes in colorectal cancer by the ESTIMATE algorithm. Next, we screened 292 tumor microenvironment-related DEGs obtained from the intersection of stromal and immune score groups. We then conducted functional enrichment analysis and the PPI network further to understand the differentially expressed genes (DEGs). We identified 15 DEGs relevant to the overall survival of CRC. Finally, six DEGs (MMP12, CHST11, ERMN, FABP4, MEOX2, TNFAIP6) were verified to be predictive of prognosis in the GSE17538 and GSE161158 data sets and verified the protein expression levels in the HPA database. Finally, we analyzed the relationship between the expression of the identified DEGs and the immune infiltration of the CRC tumor microenvironment.

It has been reported that the tumor microenvironment (TME) has large correlations to the occurrence, evolution, and prognosis of colorectal cancer. TME provides an interaction place for the immune system and tumor. TME plays an important role in tumor occurrences and spreads. Therefore, the recognition of DEGs in the CRC microenvironment might help us formulate a more sensible management and treatment plan for CRC patients. Stromal cells and immune cells are the chief elements of the non-tumor component in TME, mainly in the evolution of cancers. ESTIMATE can compute stromal and immune scores to speculate tumor purity based on single-sample gene set enrichment analysis. The ESTIMATE algorithm, has been deployed in the researches of cutaneous melanoma^20^, lung cancer^21,22^, breast cancer^23^, ovarian cancer^24^, endometrial cancer^25^, renal cell carcinoma^26,27^, bladder cancer^28^, pancreatic adenocarcinoma^29^ and osteosarcoma^30^, which shows good precision and practicality.

Our study first explored the relationship between stromal/immune cells scores and clinical characteristics in American CRC patients. The results indicated that immune scores in American CRC patients decreased with increasing stage and tumor metastasis. Besides, we found that stromal scores increased gradually as the T stage increased. The results were consistent with previous studies, which have indicated that lower immune scores were significantly associated with malignant progression in adrenocortical carcinoma^9^ and osteosarcoma^30^.

Then we analyzed 292 differentially expressed genes with 290 upregulated and 2 downregulated genes. The GO analysis indicated that DEGs were mainly concentrated in neutrophil activation involved in immune response, leukocyte migration, secretory granule membrane, receptor-ligand activity, immunoglobulin binding, signaling receptor activator activity, and immune receptor activity. The KEGG pathways illustrated that they were mainly enriched in cytokine- cytokine receptor interaction, phagosome, and chemokine signaling pathway. Cytokines are expressed primarily by immune cells and tumor cells in the tumor microenvironment. They are also information carriers between tumors and immune cells, which affect the changes in tumor immunity^31^. Enrichment analysis found that most of the differential genes play the role of cytokines and cytokine receptors. In the future, we can explore the therapeutic direction of tumor immunotherapy by elucidating the mechanism of cytokine action. The results manifested that they all played critical roles in the microenvironment and the progression of tumors. The module analyses indicated that they were mainly correlated with humoral immune response, dendritic cell chemotaxis, neutrophil chemotaxis, peptide ligand-binding receptors, cAMP signaling pathway neutrophil degranulation, antigen processing and presentation, hematopoietic cell lineage, lymphocyte activation, and immunoregulatory interactions between a Lymphoid and a non-Lymphoid cell. It has been reported that cAMP signaling is significantly related to cancer, and its targeting has shown specific antitumor effects^32^. The results further illustrated that these DEGs were closely associated with TME and immune response.

Moreover, we identified 15 prognosis-related genes by conducting survival analysis of the 292 DEGs. Then we verified 6 TME-related DEGs were associated with the overall survival rates of CRC patients based on the GSE17538 and GSE161158 data sets. And among these genes, the higher expression of MMP12 predicts a better prognosis, while CHST11, ERMN, FABP4, MEOX2, and TNFAIP6 predicted worse prognosis.

Matrix metalloproteinase 12 (MMP-12), also known as metalloelastase, is mainly expressed in macrophages^33^. Studies have shown that MMP-12 has a protective effect on CRC^34^. The expression of MMP-12 will reduce the expression of VEGF (vascular endothelial growth factor) and inhibit tumor angiogenesis, thereby increasing the survival rate of patients with colorectal cancer^35,36^. This study shows that the expression of MMP-12 is related to the infiltration of immune cells in the CRC microenvironment, which provides a new idea for the development of effective treatments for selective targeting of MMP12 in the future. Fatty acid-binding protein 4 (FABP4) has been reported to directly or indirectly regulate the growth and invasion of colorectal cancer through other channels^37^.

The other four candidate genes are CHST11, ERMN, MEOX2, and TNFAIP6. The expression of chondroitin sulfate (CS) predicts the poor prognosis of many human cancers^38–40^. However, a recent study has shown that chondroitin sulfate supplements can reduce the risk of CRC^41^. Carbohydrate sulfotransferase 11 (CHST11) is a crucial enzyme in the biosynthesis of chondroitin sulfate (CS). According to research findings, the expression level of CHST11 is significantly correlated with the progression of a variety of tumors^42,43^. Studies have shown that CHST11 is a new gene that can effectively detect CRC in cell-free DNA^44^. However, the role of CHST11 in the occurrence and development of colorectal cancer is not yet clear. This study showed that the high expression of CHST11 was related to the poor prognosis of CRC patients. There are few studies on ERM-like protein (ERMN) in tumours. A recent study showed that ERMN was connected to the Wnt pathway and might be a candidate gene that drives hepatoblastoma^45^. Mesenchyme Homeobox 2 (MEOX2) is related to the malignant progression of various tumors^38,46^, but its specific role in colorectal cancer has not been studied. A study suggests that MEOX2 may play a role in the carcinogenic process of colon adenocarcinoma^47^, but the specific mechanism is still unclear. TNFα-stimulated gene-6 (TNFAIP6) plays a vital role in the prognosis of various tumors^48,49^, but few studies on the specific mechanism of TNFAIP6 in the progression of CRC. However, we are particularly interested in TNFAIP6 among these six prognostic-related genes. TNFAIP6 is a potential marker of active inflammatory bowel disease^50^, and inflammatory bowel disease is one of the risk factors for colorectal cancer. Therefore, it is necessary to clarify further the potential biological relationship between TNFAIP6 and colorectal cancer. We also used the HPA database to verify the encoded proteins expression levels of the genes.

We also analyzed the correlation between the TME -related prognostic DEGs (CHST11, ERMN, FABP4, MEOX2, TNFAIP6, and MMP12) and immune cell infiltration. The results indicate that these DEGs may regulate the immune microenvironment by affecting various immune cell infiltration levels. However, the DEGs are identified by splitting samples based on the level of immune and stromal scores estimated by the ESTIMATE algorithm. Therefore, it is also possible that the genes are expressed by the infiltrating cells themselves. In tumors, immune cell infiltration is very complicated, which requires further in-depth research.

Our research may provide new ideas for analyzing the complex relationship between CRC and its tumor microenvironment. However, this study also has some limitations. First of all, the clinical information in the TCGA database is not too complete, such as the lack of grade data for colorectal cancer patients and follow-up information after surgery. This makes it impossible for us to conduct a comprehensive analysis of the prognosis of patients. Further research requires a large number of samples to verify the current results, which is also the focus of our subsequent work. Secondly, our research is based on public databases for bioinformatics analysis. At present, there is no functional experiment to verify the specific mechanism of the novel DEGs in the CRC microenvironment, so further investigations are needed to confirm in vivo and in vitro. In conclusion, we hope that this study will help discover new diagnostic and prognostic biomarkers and new treatment targets for colorectal cancer.

## Supplementary Information

Supplementary Information 1.

Supplementary Figure S1.

Supplementary Information S1.

Supplementary Information S2.

Supplementary Information S3.

Supplementary Information S4.

Supplementary Information S5.

## Data Availability

The datasets supporting the conclusions of this article are available in the TCGA database(https://portal.gdc.cancer.gov/) and GEO (https://www.ncbi.nlm.nih.gov/geo/) database.
