# Supplementary material for: Identification of tumor microenvironment-related prognostic genes in colorectal cancer based on bioinformatic methods
Source: Sci Rep. 2021 Jul 22;11:15040. doi: 10.1038/s41598-021-94541-6 (PMC8298640; doi:10.1038/s41598-021-94541-6)
Supplement: Supplementary file 1 — Supplementary Information 1. [file 41598_2021_94541_MOESM1_ESM.docx]

**S1:** Clinical data of American colorectal cancer patients in the TCGA database.

**S2:** Clinical data of colorectal cancer patients in the GSE17538 data set.

**S3:** Clinical data of colorectal cancer patients in the GSE161158 data set (The sixty-two overlapping patients are marked red).

**S4:** The 516 upregulated DEGs and 6 downregulated DEGs between high and low immune scores groups.

**S5:** The 686 upregulated DEGs and 2 downregulated DEGs between high and low stromal scores groups.

**Figure S1:** The correlation between immune/stromal scores and other clinical traits. Immune score: CRC-T (P = 0.694) (A). Stromal score: CRC- stage (P = 0.584) (B), CRC-M (P = 0.967) (C), CRC-N (P = 0.107) (D
