# Supplementary figures and images for: Identification of tumor microenvironment-related prognostic genes in colorectal cancer based on bioinformatic methods
Source: Sci Rep. 2021 Jul 22;11:15040. doi: 10.1038/s41598-021-94541-6 (PMC8298640; doi:10.1038/s41598-021-94541-6)

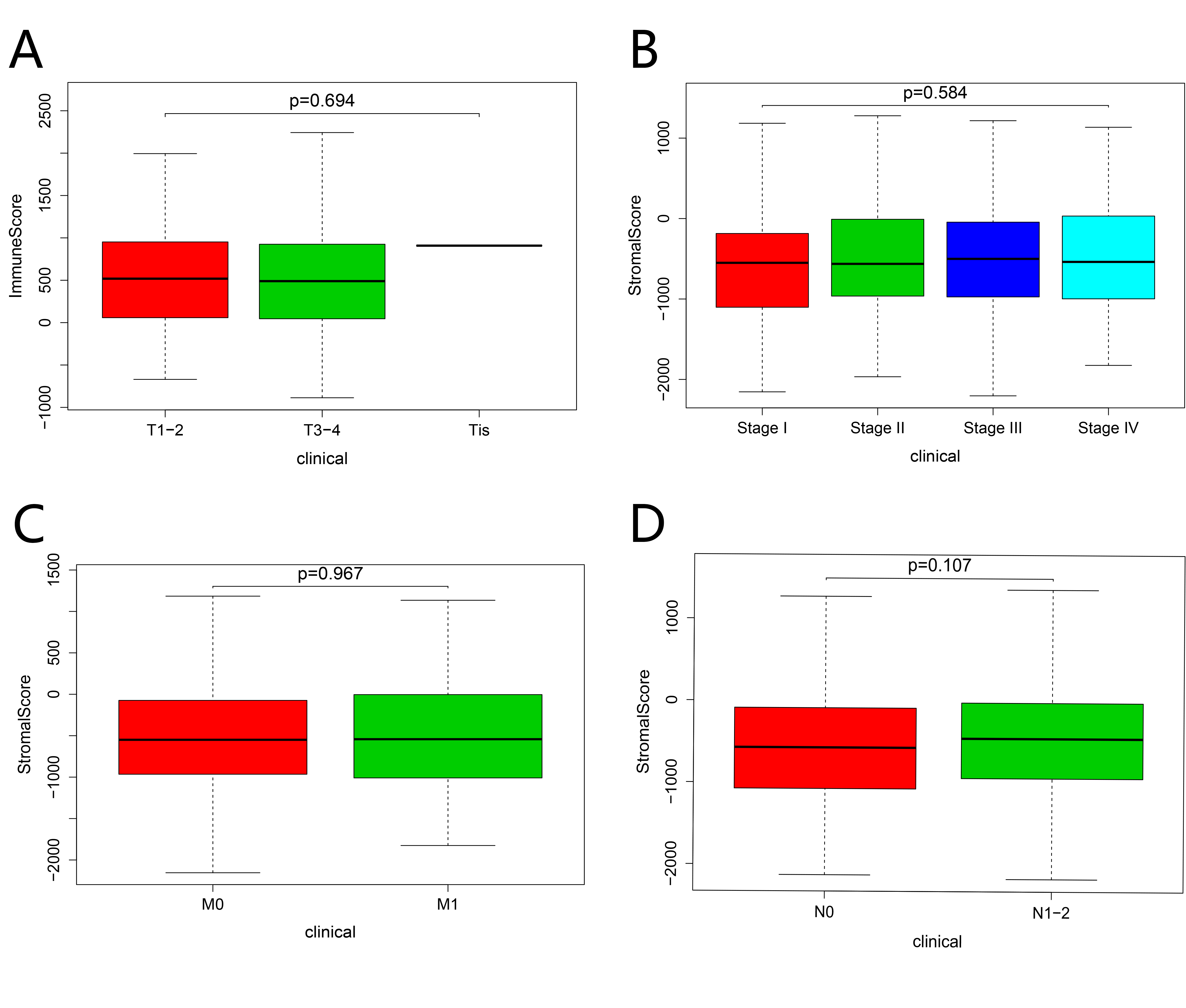

Supplement: Supplementary file 2 — Supplementary Figure S1. [file 41598_2021_94541_MOESM2_ESM.tif]
